# Cortical interneurons migrating on a pure substrate of N-cadherin exhibit fast synchronous centrosomal and nuclear movements and reduced ciliogenesis

**DOI:** 10.3389/fncel.2015.00286

**Published:** 2015-08-03

**Authors:** Camilla Luccardini, Claire Leclech, Lucie Viou, Jean-Paul Rio, Christine Métin

**Affiliations:** ^1^INSERM, UMR-S839Paris, France; ^2^Sorbonne Universités, UPMC University Paris 06, UMR-S839Paris, France; ^3^Institut du Fer à MoulinParis, France

**Keywords:** migration, cortical interneuron, cytoskeleton, primary cilium, N-cadherin, electron microscopy, videomicroscopy

## Abstract

The embryonic development of the cortex involves a phase of long distance migration of interneurons born in the basal telencephalon. Interneurons first migrate tangentially and then reorient their trajectories radially to enter the developing cortex. We have shown that migrating interneurons can assemble a primary cilium, which maintains the centrosome to the plasma membrane and processes signals to control interneuron trajectory (Baudoin et al., 2012). In the developing cortex, N-cadherin is expressed by migrating interneurons and by cells in their migratory pathway. N-cadherin promotes the motility and maintains the polarity of tangentially migrating interneurons (Luccardini et al., 2013). Because N-cadherin is an important factor that regulates the migration of medial ganglionic eminence (MGE) cells *in vivo*, we further characterized the motility and polarity of MGE cells on a substrate that only comprises this protein. MGE cells migrating on a N-cadherin substrate were seven times faster than on a laminin substrate and two times faster than on a substrate of cortical cells. A primary cilium was much less frequently observed on MGE cells migrating on N-cadherin than on laminin. Nevertheless, the mature centriole (MC) frequently docked to the plasma membrane in MGE cells migrating on N-cadherin, suggesting that plasma membrane docking is a basic feature of the centrosome in migrating MGE cells. On the N-cadherin substrate, centrosomal and nuclear movements were remarkably synchronous and the centrosome remained near the nucleus. Interestingly, MGE cells with cadherin invalidation presented centrosomal movements no longer coordinated with nuclear movements. In summary, MGE cells migrating on a pure substrate of N-cadherin show fast, coordinated nuclear and centrosomal movements, and rarely present a primary cilium.

## Introduction

Cell migration, including neuronal migration, is described as a repetitive process comprising several steps (Ridley et al., 2003; Valiente and Marín, 2010): (i) cell polarization; (ii) leading process elongation; (iii) leading process stabilization through the formation of adherent junctions and/or adhesive contacts; (iv) forward displacement of organelles by forces anchored on adhesion sites; and (v) tail retraction. The whole process involves the capability of migrating cells to polarize and to interact with cues in their environment. Although adhesive interactions play a crucial role in cell migration, their role in the migration of embryonic neurons is not fully understood (Solecki, 2012). Recent studies have demonstrated a major role of N-cadherin mediated cell-cell adhesion in the radial migration of principal cortical neurons in the mouse embryo (Kawauchi et al., 2010; Franco et al., 2011; Jossin and Cooper, 2011). They showed that N-cadherin recycling controls the adhesion of migrating neurons to the radial glia, and that N-cadherin-mediated adhesion functionally interacts with the reelin signaling pathway to control transition steps at the beginning and at the end of the radial migration. Another study in young post-mitotic cortical neurons showed that N-cadherin enrichment at one pole determines the orientation of the polarity axis of the cell, on which the nucleus and centrosome thereafter position (Gärtner et al., 2012). Whether centrosome positioning organizes cell polarity or stabilizes a polarized organization resulting from interactions with extrinsic cues, is unclear in migrating cells of which the centrosome is most often positioned in front of the nucleus (Ueda et al., 1997; Solecki et al., 2004; Higginbotham and Gleeson, 2007; Yanagida et al., 2012).

In the developing forebrain, cortical GABAergic interneurons migrate long distances from the medial ganglionic eminence (MGE) and caudal ganglionic eminence (CGE) where they originate, to the cortex where they settle among principal cells and further differentiate (Anderson et al., 1997; Wichterle et al., 1999; Nery et al., 2002; Butt et al., 2005; Wonders and Anderson, 2006; Flames et al., 2007; Fogarty et al., 2007; Miyoshi et al., 2010). Maintaining the same direction of migration during a long journey toward their final target, is thus crucial for MGE and CGE cells (Marín et al., 2010). In a previous study, we examined whether N-cadherin contributed to the long distance migration of cortical interneurons, as previously demonstrated for precerebellar neurons (Taniguchi et al., 2006). We showed that N-cadherin ablation or invalidation in MGE cells delay the tangential migration of MGE cells and alter their capacity to colonize the cortical plate (Luccardini et al., 2013). Analyses in a co-culture model showed that MGE cells with N-cadherin invalidation exhibit reduced migration speed and abnormal cell directionality because nuclear movements frequently reversed.

N-cadherin is a homophilic cell-cell adhesion protein largely expressed by telencephalic cells on which MGE cells migrate *in vivo* (Kadowaki et al., 2007). To further understand how N-cadherin contributes to the motility and directionality of migrating MGE cells, we have compared the migration of MGE cells on a pure substrate of N-cadherin, on a pure substrate of laminin that also influences cell migration and polarization (Kawauchi, 2012; Solecki, 2012), and on a substrate of cortical cells on which MGE cells exhibit their “*in vivo*-like” migration cycle. MGE cells migrated faster on the N-cadherin substrate than on any other substrate, and did not aggregate to each other. We have recently shown that the centrosome of MGE cells migrating on cortical cells can dock to the plasma membrane and assemble a primary cilium able to collect extrinsic information (Baudoin et al., 2012; Métin and Pedraza, 2014). We show here that the proportion of MGE cells with a primary cilium was strongly decreased on the N-cadherin substrate as compared with the proportion of ciliated MGE cells on the laminin substrate or on the substrate of cortical cells. Ultrastructural analyses surprisingly revealed that the mature centriole (MC) that assembles and anchors the primary cilium at the cell surface, docked to the plasma membrane at the same frequency in MGE cells cultured on N-cadherin, which rarely assemble a primary cilium, and in MGE cells cultured on cortical cells, which frequently assemble a primary cilium. In MGE cells migrating on the N-cadherin substrate, centrosomal and nuclear movements were fast and synchronous, and the centrosome preferentially localized near the nucleus. Finally, because MGE cells with an acute invalidation of N-cadherin presented centrosomal movements no longer coordinated with nuclear movements, we conclude that a pure substrate of cadherin promotes synchronous nuclear and centrosomal movements. Results moreover suggest that increased centrosomal motility does not impair the capacity of the MC to dock to the plasma membrane.

## Materials and Methods

### Animals

Pregnant Swiss mouse were purchased from Janvier Labs, and mouse embryos were produced in the animal facility of the laboratory by crossing wild type adults (Swiss, Janvier Labs). Experiments and animal care followed the European guidelines and were approved by the Ethical Committee Charles Darwin N°5 (E2CD5).

### Cell Cultures

#### Cell Cultures for Live Cell Imaging and Immunostaining

Cultures were performed on glass coverslips previously treated with nitric acid 67%, ethanol 100% and then sterilized in the oven and fixed with paraffin at the bottom of perforated petri dishes which were used as a culture chamber for the video microscopy experiments. For immunostaining, cultures were performed on glass coverslips placed in 4-well boxes.

##### Co-cultures of MGE explants on dissociated cortical cells

Cortices from wild type E13.5 mouse embryos were dissociated mechanically and the cortical cells were spread as a monolayer onto glass coverslips coated with poly-lysine and laminin (PLL/LN) as explained in Bellion et al. (2005). Cortical cells were cultured for 2 to 3 h in Dulbecco’s Modified Eagle Medium/Nutrient Mixture F-12 (DMEM/F12) (1/1) supplemented with glucose, glutamax, penicillin/ streptomycin, 4-(2-hydroxyethyl)-1-piperazineethanesulfonic acid (HEPES) buffer, N2 and B27 before placing the explants of MGE. MGEs dissected from E13.5 green fluorescent protein (GFP)-expressing transgenic mouse embryos were divided into four to six explants and placed on the substrate of dissociated cortical cells. MGEs electroporated as explained in Baudoin et al. (2012) with a plasmid encoding the pericentrin-AKAP-450 centrosomal targeting (PACT) domain of pericentrin fused to the mKO1 fluorochrome (*pCAG-PACT-mKO1*) or with a plasmid encoding the LifeAct-RFP construct (*pCAG-LifeAct-RFP*, Ibidi) were cultured for 2–4 h for recovery before placing them in culture. To analyze centrosomal movements in cadherin invalidated MGE cells, MGE explants were co-electroporated with *pCAG-EGFP-N-cad(t)* and *pCAG-PACT-mKO1* plasmids as explained in Luccardini et al. (2013). Co-cultures were maintained in the culture medium described above for 24 h before time-lapse imaging.

##### Cultures of MGE explants on a protein substrate

Clean and sterile glass coverslips were coated with PLL/LN to prepare the laminin substrate. Coverslips were coated with the extracellular domain of N-cadherin fused to the human Fc receptor protein (Lambert et al., 2000) as explained in Luccardini et al. (2013) to prepare N-cadherin substrate. Briefly, coverslips were incubated overnight at 4°C with 4 μg/ml poly-L-lysine (Sigma) and 4 μg/ml goat anti-human Fc antibody (Jackson ImmunoResearch). Coverslips were then washed in borate buffer and incubated with 1 μg/cm^2^ purified N-cad-hFc chimera protein for 3 h at 37°C. To prepare N-cadherin/laminin substrate, 4 μg/ml Laminin (Sigma) was added to the PLL and goat anti-human Fc antibody. GFP-expressing MGE explants dissected from E13.5 transgenic mouse embryos, electroporated or not with a plasmid encoding the PACT-mKO1 fusion protein, were placed on the protein substrate, and cultured 2–24 h before imaging.

#### Cultures for Electron Microscopy

##### Co-cultures of MGE explants on cortical axons

Co-cultures were performed on plastic coverslips coated with PLL/LN as explained in Baudoin et al. (2012). Because MGE cells cannot be identified by fluorescent markers in co-cultures destined to Electron Microscopy (EM) studies, they were cultured on cortical axons on which they were identified by their morphology. On this substrate, MGE cells exhibit the same migration cycle as on dissociated cortical cells (Bellion et al., 2005). Cortical explants dissected from E13.5 wild type mouse embryos were cultured for 3–5 days in DMEM/F12 (1/1) supplemented with glucose, glutamax, penicillin/streptomycin, HEPES buffer, N2 and B27. When long and numerous axons extended away from cortical explants and covered most of the surface surrounding explants, the MGE was then dissected from E12.5 wild type mouse embryos and cut equally into four to six explants. Explants were placed at the tip of corticofugal axons and cultured for 36–48 h, in order to observe the migration of numerous MGE cells on cortical axons. Co-cultures were then fixed in 1% paraformaldehyde, 1% glutaraldehyde in 0.12M PB/ 0.33 M sucrose at 4°C.

##### Cultures of MGE explants on a N-cadherin-Fc biomimetic substrate

Plastic coverslips (Thermanox) coated with the N-cadherin substrate were prepared as explained for glass coverslips (see above). The MGE dissected from E12.5 wild type mouse embryos were cut in half and deposited on a coverslip. After 24 h in culture in the DMEM/F12 culture medium (see above), cultures were fixed as described above in 1% paraformaldehyde, 1% glutaraldehyde in 0.12M PB/ 0.33 M sucrose at 4°C.

### Electron Microscopy

#### Preparation of Ultra-Thin Sections

Cultures were post-fixed in 2% Osmium tetroxide (OsO_4_) and contrasted with 1% uranyl acetate in maleate buffer. After dehydration in graded series of ethanol, cultures were transferred in araldite/ethanol (1/1) for 2 h and then overnight in 100% araldite. Small blocks with individual MGE cells migrating on either cortical axons or the N-cadherin substrate were isolated from the rest of the culture under a binocular microscope. These small regions were then embedded in a capsule of araldite with the plastic coverslip oriented parallel to the surface of the capsule. Ultra-thin sections (60 nm), parallel to the plastic coverslips, were collected on copper grids (Maxtaform Finder type H6, 200 mesh, Ted Pella Inc., Redding, CA, USA). Sections were contrasted with lead citrate solution.

#### Data Analysis

We selected several explants cultured on N-cadherin, which were surrounded by numerous migrating MGE cells (16 explants). An average of 6–11 grids were prepared with each culture. A total of 294 cells showing at least one centriole (30 grids, four independent cultures) were imaged, at minimum, at two different magnifications (×2000, ×20,000) with an ORIUS CCD camera (Gatan). In co-cultures performed on cortical axons, the density of MGE cells was strongly reduced compared to the density of MGE cells observed on the N-cadherin substrate. A total of 208 cells showing at least one centriole (98 grids, 10 independent cultures) were imaged and analyzed.

For each cell we measured the distance between the nuclear front and the centrosome, we characterized the plane of section of centriole(s) (transverse, longitudinal, oblique), and noted when centrioles were located within the leading process. The anchoring of the MC to the plasma membrane and the presence of ciliary structures (primary cilium, ciliary vesicle) at its distal end was also noted.

### Time-lapse Recording

Time-lapse recording started 2–24 h after placement of explants on the laminin or N-cadherin substrate, and 24–36 h after placement of explants on the dissociated cortical cells. For time-lapse recording, the culture medium was replaced by a culture medium without phenol red and with an increased concentration of Hepes buffer (20 mM instead of 10 mM). Co-cultures and cultures were imaged on an inverted microscope (Leica CTR 4000) equipped with a spinning disk (Roper Scientific, Trenton, NJ, USA) and a QuantEM 512SC camera (Photometrics). Cells were recorded using an X63 immersion objective, every 3 min, for an average of 4–5 h. Acquisitions were controlled using Metamorph software (Roper Scientific, Trenton, NJ, USA).

#### Analyses of Recorded MGE Cells

Three independent culture experiments performed on each substrate of migration (dissociated cortical cells, laminin, N-cadherin, and N-cadherin/laminin) were analyzed in the present study. In experiments on cortical cells and N-cadherin, six to eight MGE cells exhibiting a correct expression pattern of PACT-mKO1 were selected for centrosomal and nuclear movement analysis in each experiment. In addition to its centrosome localization, the PACT construct was distributed throughout the cytoplasm and allowed for the identification of nuclear boundary. In experiments on laminin and N-cadherin/laminin, at least 15 cells were tracked in each experiment. Cell tracking was performed with either Metamorph or ImageJ (MTrackJ, NIH, USA).

#### Statistics

Statistical analyses were performed using XLSTAT software. Mean values for a parameter in two independent samples were compared using the Student *t*-test. Distributions into classes were compared by non-parametric statistical tests: the test of Mann-Whitney for two classes, and of Kolmogorov-Smirnov for five classes.

#### Immunostaining

Cultures were fixed with 4% paraformaldehyde in 0.12M phosphate buffer/0.33 M sucrose at 4°C. Cells were incubated for 2 h with a blocking solution containing 0.25% Triton, 10% normal goat serum, 2% bovine serum albumin (BSA) in phosphate buffer saline (PBS). The following primary antibodies were used: mouse anti-Arl13b (1:1000; UC Davis/NIH NeuroMab Facility), rat anti-Tubulin YL1/2 (1:2000; abcam). Appropriate Alexa Fluor dye-conjugated secondary antibody (1:500; Invitrogen) was used to detect primary antibodies. Bisbenzimide (1:5000) was used for fluorescent nuclear counterstaining. Culture were mounted in Mowiol-Dabco and observed using an upright fluorescent microscope at X100 magnification (DM6000; Leica).

## Results

**Figure 1:**
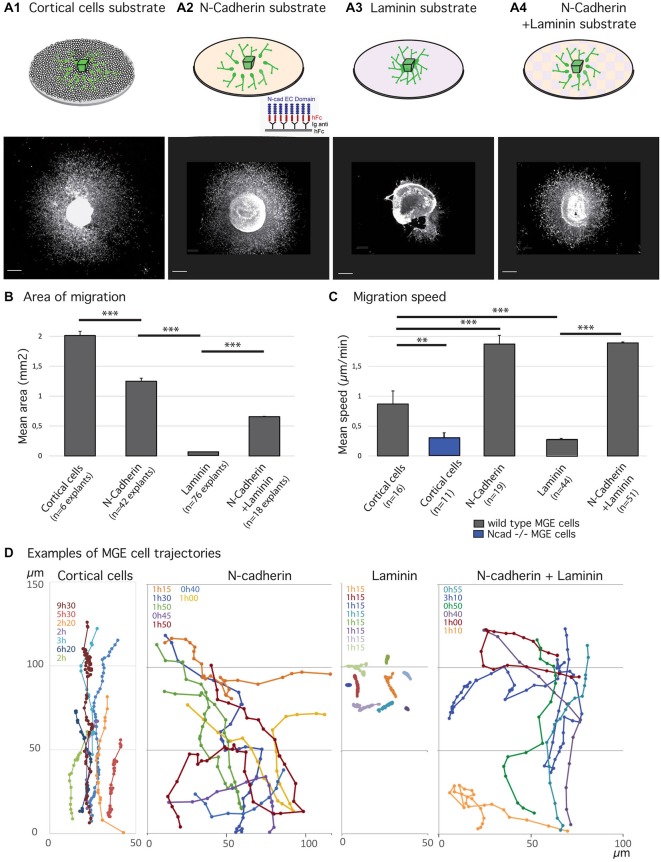
**Migratory behavior of medial ganglionic eminence (MGE) cells on various substrates of migration. (A1–B)** Analysis of migration in fixed preparations. Schemes **(A1–A4)** illustrate co-culture and culture models compared in the present study. Co-culture of MGE explants on dissociated cortical cells **(A1)** mimics the *in vivo* situation. Biomimetic substrates are prepared on glass coverslips with the extracellular domain of N-cadherin fused to a human Fc fragment, N-cad-Fc (**A2**, see “Materials and Methods” Section), or with laminin **(A3)**, or with a mixture laminin/N-cad-Fc (**A4**, see “Materials and Methods” Section). Pictures below schemes illustrate the typical distribution of green fluorescent protein (GFP)-expressing MGE cells after 24 h of migration. Scale bars, 200 μm. Histogram in **(B)** gives the average area of migration covered by MGE cells around their explant of origin. The largest migration areas are observed on cortical cells **(A1)** and on N-cad-Fc substrate **(A3)**. Values significantly different (**p < 0.01; ***p < 0.001). **(C,D)** Dynamic analysis of MGE cell migration using time-lapse videomicroscopy. Cells were imaged every 3 min for at least 30 min. Histogram **(C)** shows the mean instantaneous migration speed of the cell body of wild type (gray bars) and N-cadherin invalidated (blue) MGE cells on various substrates. Graphs in **(D)** are examples of cell trajectories at the front of the migration area (MGE explant up). Each cell track is shown in a specific color and the recording duration (hours, minutes) is indicated with the same color on the left upper corner. Dots indicate the position of the nucleus at each time point. MGE cells trajectories on dissociated cortical cells are rather straight and dots indicate the saltatory progression of the cell body. N-cad-Fc, alone or associated with laminin, strongly activates cell motility. On pure N-cadherin, MGE cells frequently turn or invert (green, yellow curves) their direction of migration. Cells movements on laminin are very slow. MGE cells are unbranched and form chains (see Figure 2A) in which cell bodies move alternatively forth and back. Trajectories are most often straight. On N-cadherin/laminin, trajectories are straighter than on N-cadherin (blue, orange curves).

MGE cells contribute to 70% of cortical interneurons. We have previously shown that MGE cells exhibit the same migratory behavior in 3-dimensional organotypic cortical slices and on dissociated cortical cells (Bellion et al., 2005). When a piece of the MGE proliferative zone of a E12.5/E13.5 mouse embryo is placed on dissociated E13 cortical cells (see scheme in Figure 1A1), MGE cells exit the explant and migrate away with radially oriented trajectories. Cells present a polarized morphology with the nucleus facing the explant and the leading process extending at the opposite side (Bellion et al., 2005). After 24–36 h in culture, MGE cells distribute over a large area around their explant of origin (picture in Figure 1A1). In these co-cultures, MGE cells present a saltatory migration characterized by coordinated nuclear and centrosomal movements. The centrosome migrates first toward the cell front and then, the nucleus translocates toward the centrosome. During the nuclear resting phase, the centrosome can migrate far away from the nucleus in close association with the endoplasmic reticulum and Golgi apparatus (GA). We have recently shown that the MC of MGE cells can dock to the plasma membrane and organize a short primary cilium at the cell surface (Baudoin et al., 2012). About one third of MGE cells with the MC anchored to the plasma membrane have a primary cilium, making the MC a basal body. Plasma membrane anchoring could stabilize basal bodies and MCs, and thereby enhance the stabilization of MGE cell polarity.

We had previously shown that *N-cadherin* ablation and cadherin functional invalidation in MGE cells reduced cell motility and impaired cell polarization, identifying N-cadherin as an important cell-cell adhesion protein that controls the migration of cortical interneurons *in vivo* (Luccardini et al., 2013). Accordingly, MGE cells cultured on a biomimetic substrate of N-cad-Fc that consists of the extracellular domain of N-cadherin attached to a glass coverslips by the Fc receptor (Lambert et al., 2000) migrated actively. These observations confirmed that N-cadherin plays a unique role to control the migration of MGE cells as the extracellular domain of  N-cadherin binds and activates N-cadherin receptors (Lambert et al., 2000). In particular, the motility of MGE cells was increased and the outgrowth of their leading process stimulated (Luccardini et al., 2013). Here, we first compared the motility and directionality of MGE cells cultured on a pure substrate of N-cadherin and on a pure substrate of laminin that binds integrin receptors and also controls cell polarity (Etienne-Manneville and Hall, 2003).

### Migratory Behavior of MGE Cells on Pure Substrates of N-Cadherin or Laminin

**Figure 2:**
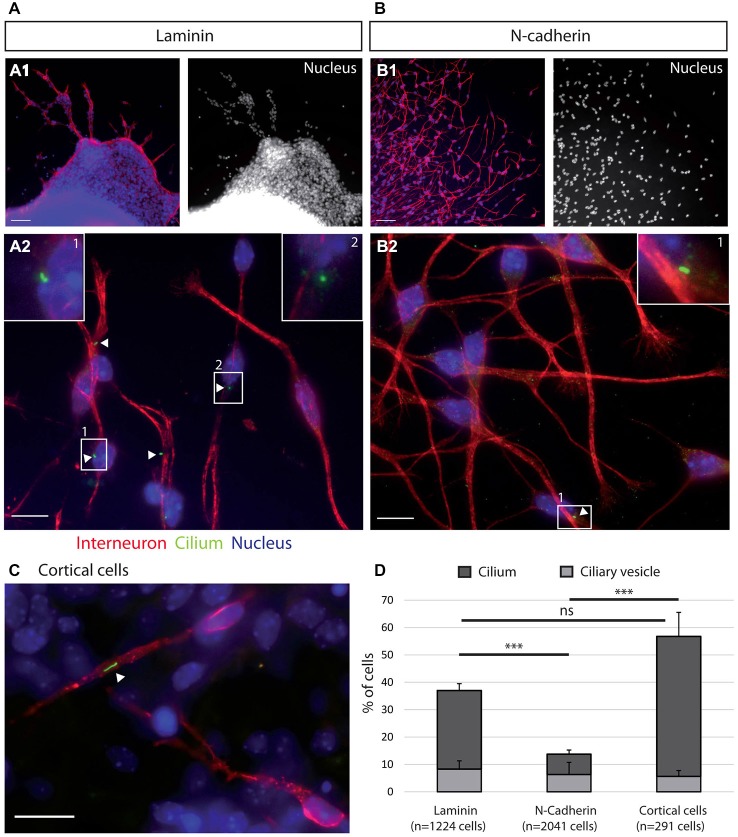
**Ciliogenesis in MGE cells depends on the substrate of migration. (A–C)** Cells that migrated away from MGE explants on a glass coverslips coated with either laminin **(A)** or N-Cad-Fc **(B)** were immunostained for the ciliary protein Arl13b (green, white arrow heads) and for tyrosinated tubulin, a marker of dynamic microtubules (red). **A1** (resp. **B1**) are low magnification views of the cultures illustrated in **A2** (resp. **B2**). MGE cells migrating on cortical cells **(C)** were electroporated with LifeActRFP plasmid (red) and immunostained for the ciliary protein Arl13b (green, white arrow head). Arl13b antibodies label either small dots [diameter <0.5 μm, see enlarged insert 2 in panel (**A**)] or longer primary cilia [length > 0.5 μm, see enlarged inserts 1 in panels (**A,B**)]. Nuclei are labeled with DAPI (blue). Scale bars: 100 μm **(A1,B1)**, 10 μm **(A2,B2)**.** (D)** Percentage of ciliated MGE cells on laminin, N-cadherin and in co-cultures. Bars show the percentages of MGE exhibiting an Arl13b positive dot (“ciliary vesicle” in legend, light gray) and a primary cilium (Arl13b positive structure longer than 0.5 μm, dark gray). Error bars represent standard error of the mean (SEM). Mean values were compared using a *t*-test (***p < 0.001; ns, non significantly different).

As already reported (Luccardini et al., 2013), MGE cells migrate much faster on the biomimetic substrate of N-cadherin than on the “*in vivo*-like” substrate of cortical cells (Figure 1). However, frequent directional changes and polarity reversals prevented MGE cells cultured on a pure N-cadherin substrate from colonizing larger areas than MGE cells cultured on cortical cells (Figures 1B,D). On a pure laminin substrate, in contrast, MGE cells migrated for very short distances around their explant of origin. After 24 h in culture, migrated MGE cells aggregated to each other to form short chains in which cells presented small amplitude forwards and backwards gliding movements (Figure 2A). When N-cad-Fc was added to the laminin substrate, MGE cells distributed over a significantly larger area that nevertheless remained inferior to the area covered by MGE cells on the pure N-cadherin substrate (Figure 1). On the mixed laminin/N-cadherin substrate, MGE cells showed fast migration speed and their trajectories were straighter than on N-cad-Fc (Figure 1C). On the N-cadherin/laminin substrate, neighboring MGE cells transiently migrated along each other and some of them exhibited polarity reversals (see yellow track in Figure 1D). As a consequence, the migration area of MGE cells was slightly but significantly reduced when compared to the migration area of MGE cells on a pure N-cadherin substrate (Figure 1B).

The morphologies of MGE cells and their spatial distribution strongly differed on the pure substrates of N-cadherin and of laminin (Figures 2A,B). Cells migrating on laminin exhibited bipolar shapes with short unbranched leading and trailing neurites whereas cells migrating on the pure N-cadherin substrate extended several long neurites at cell front and a thinner trailing process. On laminin, migrating MGE cells adhered to each other on their whole length. After 1 day in culture, they preferentially formed chains, with the exception of a few isolated cells. On N-cadherin, MGE cells moved on each other but never aggregated.

Cell dynamics and cell interactions appeared thus opposite on the pure substrates of N-cadherin and laminin. Cell motility was increased on the substrate of pure N-cadherin and decreased on the substrate of pure laminin. Cell-cell contacts were strengthened on laminin, and weakened on N-cadherin. Cell contacts moreover influenced MGE cell trajectories and the size of the area colonized by migrating MGE cells.

### Primary Cilium Frequency in MGE Cells Migrating on N-Cadherin and on Laminin Substrates

MGE cells migrating on a pure N-cadherin substrate or on a pure laminin substrate, frequently reversed their direction of migration. We have proposed that the primary cilium protruding at the cell surface could help stabilizing the centrosome (Baudoin et al., 2012; Métin and Pedraza, 2014). We thus immunostained the primary cilium in MGE cells migrating on those pure protein substrates. Immunostaining revealed that a primary cilium was much less frequent on MGE cells migrating on a N-cadherin substrate than on MGE cells migrating on a laminin substrate (Figure 2). In contrast, the primary cilium was observed at similar frequencies on MGE cells migrating on the laminin substrate and on the “*in vivo*” like substrate of cortical cells (Figures 2C,D). This result shows that adhesion proteins distributed on the substrate of migration of MGE cells directly influenced the formation and/or the stabilization of their primary cilium, and that N-cadherin alone was unable to promote the assembly and/or the stabilization of the primary cilium. In contrast, we did not correlate the presence of a primary cilium with the capacity of migrating MGE cells to maintain a fixed polarity.

**Figure 3:**
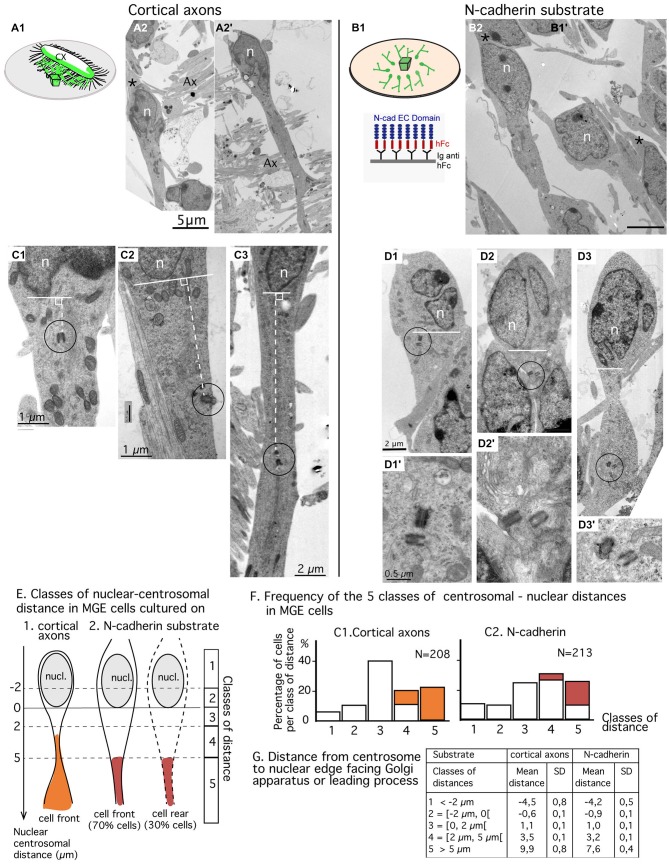
**Comparison of centrosomal position in MGE cells migrating on cortical axons vs. N-cadherin substrate using electron microscopy (EM) on ultrathin sections. (A1–B2)** MGE cells were cultured on either cortical axons **(A2,A2′)** as schematized in (**A1**) or N-cad-Fc **(B2)** as schematized in **(B1)**. On these low magnification views of ultrathin sections, migrating MGE cells are polarized. Nuclei (n) are elongated on the polarization axis. A large process, likely the leading process, extends at one pole of the nucleus. No process **(A2′)** or a thin process resembling a trailing process (black stars in **A2,B2**) is observed at the opposite pole of the nucleus. Scale bars: 5 μm in **(A2,B2). (C1–D3′)** Electron micrographs of MGE cells cultured on cortical axons **(C1–C3)** or on N-cad-Fc **(D1–D3′)** illustrate cells with different nucleus-centrioles distances: centrioles less than 2 μm in front of the nucleus **(C1,D1)**, more than 5 μm away from the nucleus **(C3,D3)** or between 2 and 5 μm **(C2,D2)**. The centriole-nuclear distance was measured as shown in panels **(C1–C3)** (white dotted line). **(D1′–D3′)** are enlarged views of the encircled centriole pair in panels **(D1–D3)**, respectively. Same scale in **(D1–D3)**, and in (**D1′–D3′)**, respectively. **(E–G)** Quantitative analysis. Scheme E defines the five classes of nuclear centrosomal distances used in the present study. The nuclear membrane facing the Golgi apparatus (GA) or the leading process defines the origin of measurements (0). Distances measured away from the nucleus, on the polarity axis of the cell, are positive. Negative centrosomal-nuclear distances were subdivided into two classes (1 and 2), whereas positive centrosomal-nuclear distances were divided into three classes (3–5). In cultures on N-cad, MGE cells with the centrosome located at the nuclear rear (about 30% of MGE cells, see text) were not distinguishable from cells with the centrosome located at nuclear front. Histograms in F show the proportion of MGE cells in each class of nuclear-centrosomal distance in co-cultures on cortical axons **(C1)** and in cultures on N-cad **(C2)**. Orange and red bars identify cells in which the centriole was located in a process or in a cytoplasmic swelling, as illustrated in **(E). (G)** Table indicates the mean nuclear-centrosomal distance and the standard deviation in each class of distance.

The primary cilium is assembled at the cell surface by the MC that docks to the plasma membrane. In order to determine whether the low frequency of primary cilia on MGE cells migrating on the pure N-cadherin substrate could result from a change in the subcellular positioning of the MC, we analyzed by EM the localization of the centrioles, especially the MC, in MGE cells migrating on the pure N-cadherin substrate. As a reference, the subcellular positioning of centrioles was analyzed in MGE cells migrating on the “*in vivo*-like” substrate of cortical axons (Figure 3).

### Subcellular Localization of the Centrosome in MGE Cells Migrating on a N-Cadherin Substrate

Ultrathin sections were performed parallel to the surface of the coverslip in the plane of migration of MGE cells. Sections that comprised the cell body of MGE cells generally comprised a significant portion (up to 15 μm) of the neuritic processes extending from the cell body (Figures 3A2,A2′). A trailing process, much thinner than the leading process, was sometimes observed at the rear of the nucleus (black star in Figure 3A2). Depending on the phase of the migration cycle, the nuclear region, the cytoplasm and the leading process were either in continuity (Figure 3A2) or separated by constricted regions that identified the rostral swelling comprising the centrosome and the GA (Figure 3A2; Métin et al., 2008; Valiente and Marín, 2010). Our previous analyses (Bellion et al., 2005; Luccardini et al., 2013) showed that the GA was positioned at cell front in almost all MGE cells cultured on cortical axons.

MGE cells cultured on N-cad-Fc showed either a polarized morphology with a leading and a trailing process that differed in thickness and length (Figure 3B2) or a complex multipolar morphology (not illustrated). Multipolar MGE cells were few in our sample. The size of the cell body was increased on N-cadherin and a clear constriction between the nuclear compartment and the rostral cytoplasmic compartment was less frequently observed. The GA was most often located in the same compartment as the nucleus. Our previous studies showed that the GA was located at the rear of the nucleus in 30% of MGE cells cultured on N-cad-Fc (see histogram of Figure 1G in Luccardini et al., 2013).

We analyzed the centrosomal-nuclear distance in MGE cells (*n* = 213) cultured on N-cad-Fc and in MGE cells (*n* = 208) cultured on cortical axons as illustrated in Figures 3C1–C3. We defined 5 classes of distances between the centrosome and the nuclear edge facing the leading process, or the GA when the leading process was difficult to identify in ultrathin section (Figure 3E). In the present study, the nuclear edge facing the leading process or the GA was considered as the nuclear front in 100% of MGE cells migrating in cortical axons, and in only 70% of MGE cells cultured on N-cadherin. It was considered as the nuclear rear in the remaining 30% of MGE cells cultured on N-cadherin.

Surprisingly, the centrosome of MGE cells cultured on N-cad-Fc was less frequently located close to the nucleus (less than 2 μm away) as compared to the centrosome of MGE cells cultured on cortical cells (24% compared to 40%, Figure 3F). On N-cad-Fc, the centrosome was most often located at a distance between 2–5 μm from the nucleus, either at nuclear front or rear. Interestingly, in MGE cells cultured on cortical axons, the centrosome was able to escape the small perinuclear region and migrated into the leading process. Accordingly, the longest nuclear-centrosomal distances were observed in MGE cells cultured on cortical axons (mean value, 9.9 μm, table in Figure 3G) rather than in MGE cells cultured on N-cad-Fc (Table in Figure 3G, mean value 7.6 μm, significantly different by a *t*-test, *p* = 0.0053). These results show that the centrosome of MGE cells migrating on pure N-cadherin distributed in a perinuclear domain significantly larger than in MGE cells cultured on cortical axons. However, on N-cadherin, the cytoplasm rarely formed a rostral swelling. We then examined whether the distribution of the centrosome in a rather large cytoplasmic compartment surrounding the nucleus affected the frequency to which the MC docked to the plasma membrane.

### Frequency of MC Docking to the Plasma Membrane in MGE Cells Migrating on N-Cadherin

**Figure 4:**
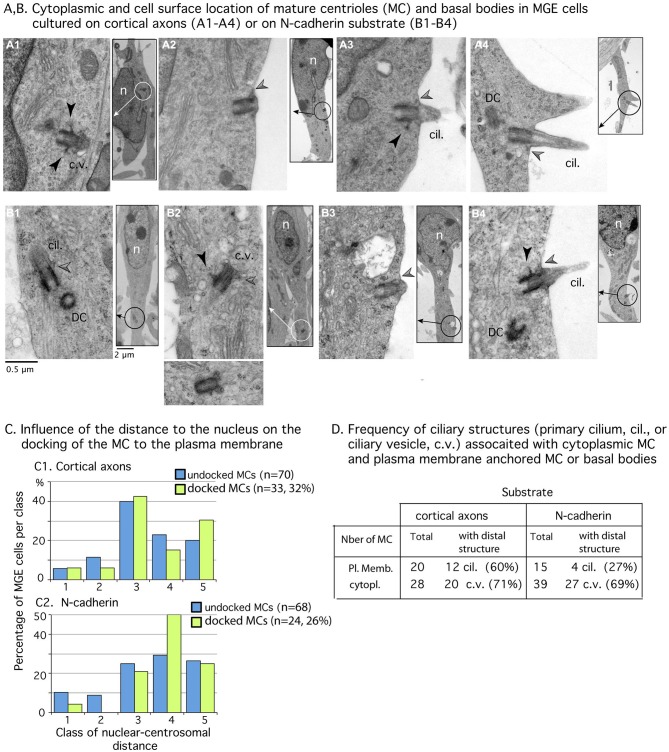
**Mature centriole (MC) subcellular localization and ciliogenesis in MGE cells cultured on cortical axons or N-cadherin. (A1–B4)** Panels show examples of MC with cytoplasmic localization **(A1,B1,B2)** or MC docked to the plasma membrane **(A2–A4,B3,B4)** in MGE cells cultured on cortical axons **(A1–A4)** and N-cad-Fc **(B1–B4)**. Inserts on the right side of panels are low magnification views of cells to localize the MC (circle). MC sectioned longitudinally show lateral appendages (black arrow heads) and/or distal appendages (gray arrow heads) which attach ciliary vesicles to the MC **(B1,B2)** or the MC to the plasma membrane **(A2–A4,B3,B4)**. The cilium is often attached to a basal body decorated with a long lateral appendage **(A3,B4)**. Same scale for all high magnification pictures and inserts (see **B1**). Cil., primary cilium; c.v., ciliary vesicle DC, daughter centriole; n, nucleus. **(C)** Histograms compare in MGEs cultured on either cortical axons or N-cad-Fc, the distribution of cytoplasmic MC (blue bars) and of MC docked to the plasma membrane (green bars) in the different classes of nuclear centrosomal distances (see Figure 3E). **(D)** Table gives the number of MC observed in the cytoplasm or docked to the plasma membrane, which exhibited a clear ciliary structure (primary cilium, cil.; ciliary vesicle, c.v.). Results confirm that primary cilia are less frequent on MGE cultured on N-cad-Fc than on cortical axons.

The MC was detected in both MGE cells cultured on N-cad-Fc (*n* = 92) and MGE cells cultured on cortical axons (*n* = 103). In ultra-thin sections, the MC was sometimes accompanied by a daughter centriole (Figures 4A4,B1,B4). Most often, the MC was isolated and identified by the presence of lateral appendages and/or distal structures such as a large vesicle or a primary cilium (Figures 4A1,A3,A4,B1,B2,B3). In some cells, the MC accumulated small vesicles at its distal end (Figures 4A1,B2). In MGE cells cultured on N-cad-Fc as well as on cortical axons, the MC was able to dock to the plasma membrane (Figures 4A2,B3). We counted very similar proportion of MC or basal bodies anchored to the plasma membrane in MGE cells migrating on either N-cad-Fc or cortical axons (26% and 32%, not significantly different by a test of Mann-Whitney, *p* = 0.67, Figure 4C).

We then examined whether the MC docked to the plasma membrane in a particular subcellular compartment. Analyses revealed that the centrosome could dock to the plasma membrane at any distance from the nucleus. Nevertheless, the MC most often docked to the plasma membrane in its preferred compartment of residency (see green bars in Figure 4C2: 50% of docked MCs were located 2–5 μm away from the nucleus). In MGE cells cultured on cortical axons, the proportion of MCs docked to the plasma membrane was slightly biased toward the leading process (Figure 4C1: 30% of docked MCs, and 20% of undocked MCs, the difference is not significant by a Kolmogorov-Smirnov test).

According to the results in MGE cells immunostained for Arl13b (Figure 2), the proportion of MCs docked to the plasma membrane and associated with a primary cilium was two folds higher in MGE cells cultured on cortical axons (55%) than in MGE cells cultured on N-cad-Fc (27%). However, in this small sample, the difference is not significant by a Mann-Whitney test. It is noteworthy that MCs located into the cytoplasm associated with a large distal vesicle or small vesicles attached to distal appendages at the same frequency regardless of the substrate of migration (69 and 71% respectively, Table **D** in Figure 4). Therefore, the ability to assemble a primary cilium at the distal end of the MC was similar in both samples, showing that the interaction of MGE cells with a N-cad-Fc substrate of migration rather did not promote primary cilium elongation on the cell surface.

### Dynamic Behavior of the Centrosome in MGE Cells Migrating on a N-Cadherin Substrate

**Figure 5:**
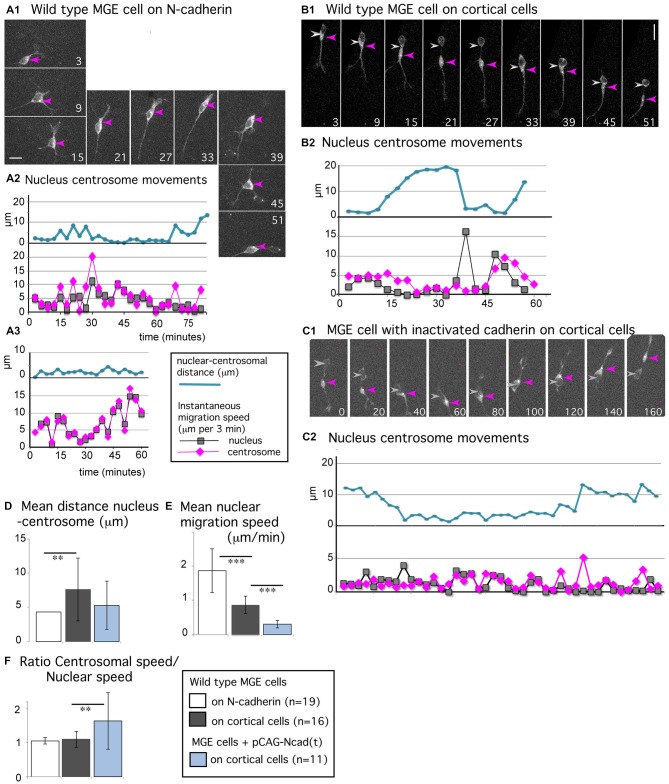
**Centrosomal movements in MGE cells migrating on a N-cadherin substrate and in MGE cells with cadherin invalidation. (A1–C2)** Time-lapse sequences in **(A1,B1,C1)** illustrate the migratory behavior of MGE cells electroporated with a pericentrin expression vector that labels the centrosome (white dot, red arrow head) and the cytoplasm (gray labeling). Pericentrin expression is also used to track the nucleus (gray arrow head). Elapsed time is indicated in minutes in panels. Scale bars, 10 μm. Curves in **(A2,A3,B2,C2)**, represent the nuclear centrosomal distance (blue curve), the instantaneous migration speed of the nucleus (gray) and centrosome (pink) in migrating MGE cells. **(A1–A3)** Migration of wild type cells on a N-cad-Fc substrate. **(B1,B2)** Migration of a wild type cell on dissociated cortical cells. **(C1,C2)** Migration on dissociated cortical cells of a cell electroporated with the dominant negative construct N-cad(t). **(D–F)** Histograms show the mean value and standard deviation of the average nuclear centrosomal distance **(D)**, of the instantaneous migration speed of the nucleus **(E)**, and of the ratio between centrosomal and nuclear instantaneous migration speeds **(F)** in wild type MGE cells monitored on N-cad-Fc (white bars), on cortical cells (gray bars) and in cadherin invalidated MGE cells monitored on cortical cells (blue bars). The nuclear speed is maximal in wild type MGE cells migrating on the pure N-cadherin substrate, and minimal in cadherin invalidated MGE cells. **p < 0.01; ***p < 0.001. Centrosomal and nuclear movements are synchronized in MGE cells migrating on the pure N-cadherin substrate; cadherin invalidation in MGE cells migrating on cortical cells disrupts the coordination between nuclear and centrosomal movements observed in wild type MGE cells.

Our EM analysis revealed that the MC of MGE cells migrating on a N-cad-Fc substrate docked to the plasma membrane at the same frequency as the MC of MGE cells migrating on cortical axons. However, the subcellular distribution of the centrosome in MGE cells migrating on a N-cadherin substrate and on cortical axons strongly differed. We thus analyzed the dynamic behavior of the centrosome in MGE cells cultured on a pure N-cadherin substrate. MGE cells were electroporated with a PACT-mKO1 construct to label the centrosome and identify nucleus position (Figure 5).

MGE cells migrating on N-cad-Fc alternated between straight and curved trajectories (Figure 1D) in correlation with their morphology. MGE cells with a bipolar shape followed straight trajectories (see panels 21–33 in Figure 5A1) whereas MGE cells with a multipolar shape continuously changed their direction of movements (see panels 3–15 and 39–51). The nucleus did not present resting phases (gray curves in Figures 5A2,A3) and the centrosome moved together with the nucleus (mean migration speed, 1.94 μm/min). The centrosome most often remained located at the same pole of the nucleus, even when the direction of migration reversed (pink arrow head in Figure 5A1, panels 39–45). The centrosome could also enter the leading process for very short duration. Nuclear centrosomal distance was maintained short (blue curves in Figures 5A2,A3, white bar in histogram Figure 5D) and the ratio between the mean instantaneous migration speed of the nucleus and of the centrosome was equal to 1 (1.05 ± 0.09, white bar in histogram of Figure 5F). In summary, the nucleus and centrosome presented fast and synchronous movements.

Synchronized centrosomal and nuclear movements are rarely observed in MGE cells migrating *in vivo* or on a complex substrate of cortical cells (Baudoin et al., 2012; Figures 5B1,B2). The nuclear progression is saltatory (see the distribution of nuclear positions in Figure 1D, and peaks of nuclear speed on gray curve in Figure 5B2), whereas centrosomal movements are progressive, with smaller peaks of migration speed and faster forward movements between peaks (pink curve in Figure 5B2). The nuclear-centrosomal distance shows large amplitude variations, ranging from 0 to 20 μm (blue curve in Figure 5B2, and gray bar in histogram Figure 5D, significantly different from the white bar by a *t*-test, *p* = 0.009). Nevertheless, centrosomal movements are coordinated with nuclear movements even if both organelles do not move synchronously. Accordingly, the ratio between centrosomal and nuclear mean instantaneous migration speeds was close to 1 in the present sample (1.1 + 0.2, gray bar in histogram Figure 5F). To further understand the contribution of cadherin mediated cell-cell adhesion in those centrosomal and nuclear movements, we electroporated MGE cells with a dominant negative construct (pCAG-N-cad(t)) that sequesters endogenous catenins and interferes with signaling pathways downstream cadherin (Taniguchi et al., 2006). As previously reported (Luccardini et al., 2013), the migration speed of the nucleus was drastically reduced in MGE cells with cadherin invalidation and cell polarity frequently reversed (Figures 5C1,C2, and blue bar in histogram Figure 5E). Surprisingly, the centrosome remained more motile than the nucleus, moving from one nuclear pole to the other. Accordingly, the ratio between centrosomal and nuclear instantaneous migration speeds increased to 1.6 (±0.8), showing that cadherin invalidation in MGE cells partially disrupted the correlation between centrosomal and nuclear movements. This result suggests that cadherin activity promotes the synchronization of nuclear and centrosomal movements, which was indeed remarkably tight in MGE cells migrating on a pure N-cadherin substrate.

## Discussion

In the embryonic brain, N-cadherin is present with other adhesive and guidance cues in the migratory pathway of MGE cells. Because we observed that N-cadherin was the most efficient protein substrate to promote the migration of MGE cells *in vitro*, we decided to further characterize the migratory behavior of MGE cells on this permissive substrate. We moreover compared the migration of MGE cells on a pure N-cadherin substrate with their migration on a pure laminin substrate. The migration speed of MGE cells was very slow on the pure laminin substrate and cells preferentially adhered to each other. On N-cadherin in contrast, contacts between MGE cells were minimal. Centrosomal movements were highly synchronized with nuclear movements. The MC remained able to dock to the plasma membrane but a primary cilium rarely formed. Therefore, cadherin signaling not only influences nuclear motility in migrating MGE cells, but moreover influences centrosomal movements and ciliogenesis.

### Migratory Behavior of MGE Cells on a Pure Substrate of N-Cadherin or of Laminin

Two major types of cell adhesion regulate the cell migration: (1) cell-cell adhesion that is controlled, among other proteins, by classical cadherins that establish homophilic interactions; and (2) cell-extracellular matrix adhesion that is controlled, among others, by integrin receptors that link extra-cellular matrix (ECM) proteins as laminin or fibronectin to the actin cytoskeleton (Kawauchi, 2012). N-cadherin is expressed by MGE cells and by cells in their migratory pathway (Kadowaki et al., 2007). We previously showed that N-cadherin loss of function in migrating MGE cells altered cell motility and cell polarity (Luccardini et al., 2013). To further characterize the role of N-cadherin mediated cell-cell adhesion in the migration of cortical interneurons, we analyzed the migratory behavior of MGE cells on a biomimetic substrate of N-cadherin and on a pure laminin substrate that activates integrins, a class of cell surface receptors involved in neural migration and cell polarity (Lawson and Burridge, 2014). In contrast to laminin on which MGE cells moved very slowly, N-cadherin stimulated the outgrowth of leading processes and the motility of MGE cells. Additional differences distinguished MGE cells cultured on laminin or N-cadherin. First, reciprocal interactions between MGE cells differed. MGE cells migrating on N-cadherin only shortly interacted and never aggregated, even if their density was high. Adhesive interactions between MGE cells were thus minimal. On the laminin substrate in contrast, MGE cells exhibited long-lasting and tight reciprocal interactions that quickly led to the formation of chains or cell aggregates. On this latter substrate of migration, MGE cells not only interacted with the protein substrate, but moreover with neighboring cells. These tight and long-lasting cell-cell interactions likely involve N-cadherin receptors, among other receptors, suggesting that the response of MGE cells to the laminin substrate was perturbated by additional and uncontrolled cell-cell interactions. By adding laminin to the N-cadherin substrate, we increased the frequency and duration of cell-cell contacts, suggesting functional interactions between integrin and N-cadherin receptors in migrating MGE cells. Second, MGE cells migrating on N-cadherin and on laminin differed by their morphologies and trajectories. On the laminin substrate, MGE cells were mainly bipolar and showed straight trajectories, whereas MGE cells migrating on N-cadherin alternated between bipolar and multipolar morphologies and showed complex trajectories. These differences in morphology suggest major differences in the organization of the cytoskeleton on the two substrates and will require further investigations.

### Influence of N-Cadherin Biomimetic Substrate on Centrosome Docking and on Primary Cilium Assembly

We have shown that the centrosome of GABAergic neurons born in the MGE of the basal telencephalon is a microtubule-organizing center that can moreover assemble a primary cilium at the distal end of the MC (Baudoin et al., 2012). An important function of the primary cilium is to anchor the centrosome to the plasma membrane (Pedersen et al., 2008; Reiter et al., 2012). In GABAergic neurons migrating a long distance from the basal telencephalon to the cortex, the MC is able to dock to the plasma membrane. It can associate with a primary cilium protruding at the cell surface (Baudoin et al., 2012; Higginbotham et al., 2012). In the cell samples that we analyzed here, the primary cilium was less frequently observed on MGE cells cultured on N-cad-Fc than on MGE cells cultured on laminin or on cortical cells. However, the proportion of MGE cells with the MC docked to the plasma membrane did not decrease when MGE cells were migrating on a pure substrate of N-cadherin. Moreover, the MC was able to dock on the cell surface at any distance from the nucleus. We observed ciliary vesicles at the same frequency in MGE cells cultured on cortical axons or on N-cad-Fc. The large ciliary vesicle observed at the distal end of MCs likely contributes to the anchoring of the MC to the plasma membrane through a mechanism involving membrane fusion (Sorokin, 1962). Therefore, the ability of the MC to assemble a ciliary vesicle and to dock to the plasma membrane was not perturbed on the substrate of N-cadherin, suggesting that the N-cadherin mediated cell-cell interaction did not allow primary cilium elongation or stabilization. This response of the primary cilium to a cell adhesion protein distributed in the environment was specific to N-cadherin since MGE cells migrating on laminin exhibited a primary cilium at the same frequency as MGE migrating with a “*in vivo like*” substrate of cortical cells.

An attractive hypothesis is that MC docking could interfere with centrosome motility. Our results do not support this hypothesis. Indeed, the centrosome moved twice faster in MGE cells cultured on N-cad-Fc than in MGE cells cultured on cortical cells, although it docked to the plasma membrane at the same frequency in both culture conditions. Even if our results argue against a role of plasma membrane docking to reducing MC motility, we cannot totally exclude this possibility. The primary cilium itself could contribute to regulate MCs motility by interacting with the substrate of migration. In agreement with our present results, we previously showed that the genetic ablation of the primary cilium in Kif3a KOs MGE cells was correlated to a slight increase in the motility of the centrosome (Baudoin et al., 2012).

Integrin receptors have been described on the primary cilium (McGlashan et al., 2006; Seeger-Nukpezah and Golemis, 2012). Adhesive interactions with the extracellular matrix could transiently stabilize the primary cilium and the basal body. Accordingly, the apical abscission of the primary cilium helps centrioles to move away from the basal lamina in progenitors of the cortical neuroepithelium (Das and Storey, 2014).

The role(s) of the primary cilium in cell migration is far from understood (Albrecht-Buehler, 1977; Katsumoto et al., 1994; Schneider et al., 2010; Métin and Pedraza, 2014). In adherent cells like fibroblasts or smooth muscle cells, the cilium has been shown to control cell directionality. The primary cilium of cortical interneurons concentrates receptors to factors in the environment that likely influence the migration of cortical interneurons (Higginbotham et al., 2012). The simultaneous monitoring of centrosomal and primary cilium dynamics shall permit to examine whether the primary cilium interferes with centriole motility in addition to collecting extrinsic signals.

### In MGE Cells Migrating on N-Cadherin, the Centrosome is Maintained at One Pole of the Nucleus

The present study revealed an unexpected behavior of the centrosome in MGE cells migrating on N-cad-Fc. In control MGE cells migrating on cortical cells the centrosome showed a progressive forward migration in front of the nucleus (Métin et al., 2008). After each nuclear translocation, the centrosome starts moving toward the leading process where it can stabilize before the next nuclear translocation (Yanagida et al., 2012). On N-cad-Fc, centrosomal movements were most often restricted to a subcellular domain located at one pole of the nucleus. This domain, that contained the GA and endoplasmic reticulum (ER) vesicles, could be large and the centrosome only rarely escaped to enter a process emerging from the cell body. The centrosome remained there, even during the time period when the cell reversed its direction of migration and when the nucleus moved alternatively forward and backward. This behavior of the centrosome is opposite to the behavior observed in N-cadherin ablated or N-cadherin invalidated MGE cells, in which the centrosome exhibited fast and large amplitude movements in the whole nuclear compartment, moving from the front to the rear of the nucleus. N-cadherin KO MGE cells frequently reversed their direction of movement and showed alternating forward and backward nuclear movements, as control MGE cells cultured on N-cad-Fc (Luccardini et al., 2013). N-cadherin ablation or invalidation disrupted the coordination between nuclear and centrosomal movements in MGE cells. On the contrary, N-cadherin activation stabilized the centrosome in a sub-region of the nuclear compartment. This important role of N-cadherin to stabilize the positioning of the centrosome in migrating MGE cells recalls N-cadherin function to control cell polarity in neural cells (Dupin et al., 2009; Gärtner et al., 2012), and to control *in vivo*, the polarized organization of cortical progenitors in the proliferative neuroepithelium (Kadowaki et al., 2007). Accordingly, the expression level of N-cadherin has also been shown to control the polarity and migration speed of glial cells in a wound-healing test (Camand et al., 2012). In MGE cells migrating on N-cad-Fc, the nuclear and centrosomal movements were highly synchronized, which was not the case in MGE cells migrating on cortical cells. The regulatory mechanisms involved in this control remain to be explored.

In conclusion, we show here that N-cadherin-mediated cell-cell interactions are efficient signals for stimulating MGE cell motility, including centrosome motility. The negative correlation between centrosomal movements and primary cilium formation observed in MGE cells migrating on N-cadherin, remembers results in MGE cells with a genetic ablation of the primary cilium (Baudoin et al., 2012). In addition, our results show that N-cadherin mediated cell-cell interactions and integrin mediated ECM-cell interactions have distinct influences on the ciliogenesis in migrating MGE cells.

## Conflict of Interest Statement

The authors declare that the research was conducted in the absence of any commercial or financial relationships that could be construed as a potential conflict of interest.
